# Opportunistic Eye Disease Screening in Mazovia, Poland: Lessons from a Local Government Program: “Good Vision for Mazovians”

**DOI:** 10.3390/healthcare13192456

**Published:** 2025-09-27

**Authors:** Agnieszka Kamińska, Olga Adamska, Maciej Kamiński, Anna Pierzak, Andrew Lockley, Szymon Rybicki, Mateusz Jankowski, Radosław Sierpiński

**Affiliations:** 1 Ophthalmology Department, Faculty of Medicine, Collegium Medicum, Cardinal Stefan Wyszynski University in Warsaw, 01-938 Warsaw, Poland; 2 Ophthalmology Department, Miedzylesie Specialist Hospital, 04-749 Warsaw, Poland; 3 Faculty of Medicine, Łazarski University, 02-662 Warsaw, Poland; 4 School of Public Health, Centre of Postgraduate Medical Education, 01-826 Warsaw, Poland; 5 Faculty of Medicine, Collegium Medicum, Cardinal Stefan Wyszynski University, 01-938 Warsaw, Poland

**Keywords:** local government unit program, eye health, screening program, health policy, prevention, vision impairment

## Abstract

**Background**: Vision loss due to chronic eye diseases remains a significant public health challenge. Early detection through screening programs may reduce the burden of vision loss. This study aimed to assess the detection rate of eye diseases (glaucoma, AMD, and diabetic retinopathy), including those newly detected during opportunistic screening and ophthalmological consultations within the local government health policy program “Good Vision for Mazovians” in Mazovia, Poland. **Material and methods**: This study is a retrospective analysis of medical data from the registry of the Ophthalmology Department of the Międzylesie Specialist Hospital in Warsaw, which implemented the local government preventive program “Good Vision for Mazovians. Data from 1812 individuals (aged 18–92 years) participating in the “Good Vision for Mazovians” preventive program were analyzed. **Results**: Most participants were female (59.7%), aged over 60, and took medications regularly (62.7%). Excluding subjects with prior diagnosis of eye conditions, the detection rate was 38 suspected cases (3.8%) of glaucoma cases, 84 suspected cases of AMD (4.6%), and 21 suspected cases of diabetic retinopathy (1.2%). Most participants had not visited an ophthalmologist in the past two years (58.6%), reported low or average knowledge of eye health, had difficulty accessing ophthalmology services in their region (57%), and identified long waiting times for appointments as the main barrier to care (83.5%). **Conclusions**: Opportunistic screening for eye diseases in populations with limited access to eye care should be considered as a method for detecting common causes of irreversible visual impairment, particularly AMD. Older adults and individuals without higher education appear to face the greatest barriers to accessing ophthalmology services and may benefit the most from targeted opportunistic screening initiatives.

## 1. Introduction

Vision loss due to chronic eye diseases remains a significant public health challenge [1]. Population aging and individual lifestyle-related behaviors contribute to the growing burden of eye diseases like glaucoma, age-related macular degeneration (AMD), or diabetic retinopathy [2,3,4]. These eye diseases are leading causes of irreversible visual impairment [1,2,3,4]. As glaucoma, AMD, and diabetic retinopathy are often asymptomatic for years and progress silently, early detection plays a crucial role in limiting progression and avoiding irreversible damage [2,3,4]. Early detection of eye diseases is a key action in reducing the burden of visual disability [1,5]. Between 2010 and 2019, global crude prevalence of avoidable vision impairment and blindness in adults aged 50 years and older did not change [5]. The reduction of age-standardized rates of avoidable blindness was not reached in the global population [5]. Early detection of eye diseases plays a crucial role in the reduction of vision impairment and blindness rates [5].

Providing access to eye care services is an important responsibility of public authorities in Poland, a country with a health system based on mandatory health insurance and publicly funded health services [6]. Each insured individual can visit a general practitioner who may refer the patient to an ophthalmologist (referral required) [6]. Moreover, there is access to private care services paid out of pocket or private health insurance [6,7]. Basic measurements of vision defects (myopia, hyperopia) can also be performed by optometrists in optical shops [7].

A nationwide cross-sectional survey among adults in Poland, performed in 2022, showed that 31.2% of adults in Poland had eye examinations over 3 years ago (24.1%) or never (7.1%) [8]. In Poland, wearing glasses or lenses and self-reported level of knowledge on eye diseases were identified as the only factors significantly associated with higher odds of eye examinations [8]. Another population-based study also showed that a low level of awareness of common eye diseases among adults in Poland [9]. Low public awareness of eye diseases and missing regular eye screening in Poland [8,9] point out the need to provide public health interventions for the early detection of eye diseases.

To bridge the gap in health services planned at the national level, Local Government Units (LGUs) in Poland may develop health policy programs on different health conditions [10,11]. Between 2015 and 2023, out of 1568 health policy programs prepared in Poland, only 41 (2.6%) programs addressed eye health [10]. LGUs can provide essential healthcare services to communities through primary healthcare centers, local hospitals, and outreach programs [10]. Opportunistic screening is a form of preventive health services that takes place “incidentally” with other activities, such as visits to public places (e.g., shopping malls, health centers, health fairs, etc.) [12]. Opportunistic screening aims to detect diseases or risk conditions that may be undetected, but which can be treated or monitored at an early stage. Previously published studies evaluated the cost-effectiveness of implementing glaucoma or diabetic retinopathy screening in primary care [13,14,15]. Opportunistic screening was used in the past for the early detection of eye diseases, like glaucoma or diabetic retinopathy [12,16]. Currently, there is also a debate on the application of artificial intelligence in eye disease screening programs [17].

“Good Vision for Mazovians” is the local government program on opportunistic screening for common eye diseases (secondary prevention) like glaucoma, AMD, and diabetic retinopathy, implemented in 2024 [18]. The program is targeted at residents of Mazovian Voivodeship, the central region of Poland, with the seat of local government authorities in Warsaw, and 5.5 million citizens. Data on LGU’s programs on opportunistic screening may provide data on program effectiveness, outcomes, and priority populations or populations experiencing health inequalities and attending this type of program to obtain health services.

This study aimed to assess the detection rate of eye diseases (glaucoma, AMD, diabetic retinopathy) within the local government health policy program in Mazovia, Poland. The secondary aim was to characterize individuals participating in opportunistic screening in areas with limited access to ophthalmological clinics.

## 2. Materials and Methods

### 2.1. Study Design and Population

This study is a retrospective analysis of medical data from the registry of the Ophthalmology Department of the Międzylesie Specialist Hospital in Warsaw (retrospective analysis of hospital database), which implemented the local government preventive program “Good Vision for Mazovians” [18]. “Good Vision for Mazovians” was an eye screening opportunity program funded by the Marshal’s Office of the Mazovian Voivodeship. The program aimed to detect eye conditions early, based on a series of preventive screening examinations and an educational campaign about eye health. As part of the program, 12 days of examinations were conducted in 9 locations (3 sites were visited twice) between March and December 2024.

The dataset used in this study was generated based on the medical records from the residents of the Masovian Voivodeship, who participated in the “Good Vision for Mazovians” program in 9 locations. The locations of the examination sites were selected to ensure access to examinations for residents of areas with limited access to ophthalmological clinics. The local government at the study site (e.g., city or municipal office) was asked to designate rooms where the team from the Ophthalmology Department of the Międzylesie Specialist Hospital in Warsaw, in collaboration with project partners, set up temporary ophthalmology offices (examination rooms and consultation rooms). The local government at the study site was asked to conduct an information campaign about the project (dates, locations, and hours of testing) to inform the local community about the possibility of testing. All interested subjects could participate in the study free of charge. No prior registration or referral was required (self-selection occurred).

Inclusion criteria: age 18 and over; willingness to participate in the study.

Exclusion criteria: age under 18 years; lack of willingness to participate in the study.

Written informed consent was collected from all the participants when registering for screening tests. The study protocol was reviewed and approved by the Ethics Committee at the Centre for Medical Postgraduate Education (decision number 16/2025 as of 12 February 2025).

### 2.2. Measures

Each subject interested in participating in the study who provided written informed consent was asked to complete a questionnaire specifying demographic data, a brief medical history including past diagnosis of eye conditions, history of eye examinations, and perception of barriers to accessing ophthalmological care. At the first stage, for each subject, the nurse measured intraocular pressure with a tonometer (Canon full auto tonometer TX-20P, Kanagawa, Japan) and measured ocular refraction using an autorefractometer (Tomey autorefractometer RC-800, Nagoya, Japan). The patient was then referred to a medical office for a fundoscopy examination using the DRSplus (Digital Retinography System) and iCare ILLUME software (CenterVue, iCare DRSplus TrueColor confocal fundus imaging system, Padova, Italy). Based on the assessment of the fundus and the physician’s decision, the physician might refer the patient for an Optical Coherence Tomography (OCT) scan (Optopol Technology, SOCT REVO-60, Zawiercie, Poland) to evaluate the retina and macula. In case of poor quality of the imaging, the scan was repeated after pharmacological mydriasis with 1% Tropicamide. Following the conducted examinations, the physician would suspect a diagnosis of glaucoma, age-related macular degeneration (AMD), or diabetic retinopathy, and the patient would receive a referral to their primary care physician for further treatment.

A patient was classified as having suspected glaucoma if they showed positive results in at least two of the three following parameters:

1. Intraocular pressure elevated above 21 mmHg, weighted with central corneal thickness correction measured in tonometry.
2. Optic nerve damage assessed in OCT scans is shown as retinal nerve fiber layer loss.
3. Reports suggesting glaucoma from Fundus Imaging System, such as an enlarged optic cup or a large cup-to-disc ratio, vertical elongation of the cup, notching or thinning of the neuroretinal rim, and nasal displacement of the vessels.

According to the European Glaucoma Society Terminology and Guidelines for Glaucoma, patients classified for further evaluation were directed to an ophthalmology outpatient clinic for applanation tonometry, gonioscopy, visual field examination, as well as confirmation of diagnosis and initiation of treatment if needed [19].

A patient was classified as having suspected age-related macular degeneration (AMD) after considering the patient’s age, reports suggesting AMD from DRSplus, and OCT scan abnormalities. Among the abnormalities were the presence of drusen, pigmentary abnormalities, RPE atrophy, RPE degeneration and dysfunction, the presence of serous or hemorrhagic retinal PED, a subretinal neovascular membrane, or a subretinal hemorrhage [20]. After receiving test results, patients were referred to an ophthalmology outpatient clinic for further diagnosis and treatment.

A patient was classified as having suspected diabetic retinopathy when presenting with typical abnormalities in fundus examination: microaneurysms, retinal bleeding, cotton wool spots, and hard exudate, venous beading, intraretinal microvascular anomalies, and retinal neovascularization, all of which were later evaluated by DRSplus. Optical coherence tomography was used to determine the presence of macular edema [21]. A suspicion of diabetic retinopathy resulted in a referral to a general practitioner for evaluation and eventual modification of diabetes therapy [22] and to an ophthalmology outpatient clinic for additional tests.

Any patient in whom eye conditions were suspected had the option to report to the ophthalmology outpatient clinic at the Międzylesie Specialist Hospital in Warsaw for the final diagnosis and treatment initiation. There were no follow-up data on the number of patients who attended the ophthalmologist for diagnosis confirmation or indicated treatment, so all cases (AMD, glaucoma, diabetic retinopathy) detected during the screening program were suspected cases. All procedures at all sites were performed by the same medical team, the same set of medical devices, and following the same standards. In this study, the detection rates (glaucoma, AMD, or diabetic retinopathy) during the examinations provided by the “Good Vision for Mazovians” were analyzed. Moreover, as some patients reported eye conditions detected in the past, a sub-analysis for those with prior medical diagnosis of eye diseases and without a history of eye diseases was presented.

Based on data from the questionnaire filled out on admission, barriers to access ophthalmological care and history of eye examinations were analyzed.

### 2.3. Statistical Analysis

Medical records from the hospital registry were coded into an anonymous database by ophthalmologists employed at the Ophthalmology Department of the Międzylesie Specialist Hospital in Warsaw. Statistical analysis was completed with SPSS v.29 (IBM, Armonk, NY, USA). The distribution of categorical variables was shown by frequencies and proportions. Categorical variables were compared using the cross-tabulation and the independent samples two-tailed chi-squared test. Statistical significance level was set at *p* < 0.05.

## 3. Results

Data on 1812 individuals (aged 18–92 years) who participated in the local government preventive program “Good Vision for Mazovians” were analyzed. Most of the respondents were females (59.7%), took medications on a regular basis (62.7%), and were aged over 60 years (Table 1). Among the participants, 41.3% had hypertension, 17.2% had heart disease, 11.8% had diabetes, and 6.6% had lung disease. Details are presented in Table 1.

Among the participants (*n* = 1812), 59.7% used glasses or contact lenses, 3.8% had ever been diagnosed by a doctor with glaucoma, 0.7% with AMD, and 0.6% with diabetic retinopathy (Table 2). Among the participants, over one-third had an eye examination more than 3 years ago, and 7.6% declared that they had never had an eye examination. Only 41.4% of participants visited an ophthalmologist in the last 2 years. Over half of respondents (57%) had difficulty accessing an ophthalmologist in their region, but 83.4% declared that there was access to an optician’s shop in their place of residence. Among those who experienced difficulties accessing an ophthalmologist, waiting time for a visit was a major barrier, indicated by 83.5% of respondents (Table 2). Only 4% of respondents declared rather good or very good knowledge about eye diseases, but 87.6% declared willingness to improve their knowledge about eye disease (Table 2).

In total, 69 suspected cases of glaucoma were detected, of which 38 (2.1% of all participants) were newly detected in participants without prior diagnosis of eye conditions, and 31 were in line with data on medical diagnosis in the past, self-reported by patients.

In screening tests performed in this study, a total of 92 suspected cases of AMD were detected (5.1%), of which 84 were newly detected suspected cases during the opportunistic screening in participants without prior diagnosis of AMD.

In screening tests performed in this study, a total of 29 suspected cases of diabetic retinopathy were detected, of which 21 were newly detected (without prior medical history diagnosis) (Table 3).

The detection rate of AMD and diabetic retinopathy increased (*p* < 0.001) with age (Table 4). Moreover, most of the AMD (*p* = 0.007) or diabetic retinopathy (*p =* 0.02) cases were detected in those with primary education, as well as those who took medications on a regular basis (*p* < 0.001). There were differences (*p* < 0.001) in the detection rate of glaucoma and AMD between research sites (Table 4).

Females, compared to males (44.5% vs. 36.8%; *p* < 0.001), more often declared that they visited an ophthalmologist in the last 2 years (Table 5). Moreover, over half of respondents aged 18–29 years or those aged 80 years and over had visited an ophthalmologist in the last 2 years. Those with higher education, compared to those without a university degree (*p* = 0.002), more often declared that they had visited an ophthalmologist in the last 2 years. Those who took medications regularly more often (*p* < 0.001) declared that they had visited an ophthalmologist in the last 2 years (Table 5). Females, compared to males (61.9% vs. 49.8%; *p* < 0.001), more often have difficulty accessing an ophthalmologist in the region where they live (Table 5). The percentage of respondents who declared difficulty accessing an ophthalmologist increased with age (*p* < 0.001). Those who took medications on a regular basis more often (*p* < 0.001) declared that they had difficulty accessing an ophthalmologist (Table 5). Those with higher education more often declared (*p* < 0.001) that there was access to an optician’s shop in their place of residence, compared to those without a university degree (Table 5). Older respondents more often (*p <* 0.001) declared willingness to improve their knowledge about eye diseases (Table 5). Moreover, those who took medications on a regular basis more often (*p* = 0.001) declared willingness to improve their knowledge about eye diseases.

The percentage of respondents who declared difficulty accessing an ophthalmologist, access to an optician’s shop, and willingness to improve knowledge about eye diseases differed (*p =* 0.001) by research site.

**Table 5:** Socio-demographic differences in perception of access to eye care in Mazovian Voivodeship, Poland (*n* = 1812).

	Have You Visited an Ophthalmologist in the Last 2 Years?—“Yes”			Do You Have Difficulty Accessing an Ophthalmologist in Your Region?—“Yes”			In Your Place of Residence, Is There Access to an Optician’s Shop Where You Can Buy Glasses or Contact Lenses?—“Yes”			Would You Like to Improve Your Knowledge About Eye Diseases?—“Yes”		
	*n*	%	*p*	*n*	%	*p*	*n*	%	*p*	*n*	%	*p*
Overall (*n* = 1812)	750	41.4		1033	57.0		1151	63.5		1158	63.9	
Variable												
Gender												
Female (*n* = 1081)	481	44.5	**0.001**	669	61.9	**<0.001**	912	84.4	0.2	969	88.7	0.09
Male (*n* = 731)	269	36.8		364	49.8		599	81.9		629	86.0	
Age												
<30 (*n* = 73)	38	52.1	**<0.001**	17	23.3	**<0.001**	64	87.7	0.1	57	78.1	**<0.001**
30–39 (*n* = 94)	31	33.0		38	40.4		84	89.4		75	79.8	
40–49 (*n* = 292)	105	36.0		142	48.6		255	87.3		243	83.2	
50–59 (*n* = 291)	100	34.4		158	54.3		245	84.2		258	88.7	
60–69 (*n* = 513)	208	40.5		334	65.1		418	81.5		467	91.0	
70–79 (*n* = 465)	222	47.7		288	61.0		377	81.1		415	89.2	
80+ (*n* = 84)	46	54.8		56	66.7		68	81.0		73	86.9	
Educational level												
Primary (*n* = 108)	43	39.8	**0.002**	67	62.0	0.07	67	62.0	**<0.001**	88	81.5	0.06
Vocational (*n* = 370)	113	35.9		223	60.3		268	72.4		334	90.3	
Secondary (*n* = 715)	281	39.3		415	58.0		615	86.0		632	88.4	
Higher (*n* = 619)	293	47.3		328	53.0		561	90.6		534	86.3	
Taking medications on a regular basis												
Yes (*n* = 1137)	507	44.6	**<0.001**	697	61.3	**<0.001**	938	82.5	0.2	1018	89.5	**0.001**
No (*n* = 675)	243	36.0		336	49.8		573	84.9		570	84.4	
Do you smoke cigarettes, use e-cigarettes, or heated tobacco?												
Yes (*n* = 306)	121	39.5	0.5	189	61.8	0.07	252	82.4	0.6	262	85.6	0.2
No (*n* = 1506)	629	41.8		844	56.0		1259	83.6		1326	88.0	
Research site												
No 1. (Bodzanów) (*n* = 159)	53	33.3	0.2	99	62.3	**<0.001**	51	32.1	**<0.001**	138	86.8	**0.001**
No 2. (Ciechanów) (*n* = 289)	113	39.1		201	69.6		250	86.5		255	88.2	
No 3. (Warsaw–Wilanów District) (*n* = 107)	41	38.3		47	43.9		96	92.9		83	77.6	
No 4. (Legionowo) (*n* = 108)	41	38.0		38	35.2		101	93.5		93	86.1	
No 5. (Ostrołęka) (*n* = 148)	67	45.3		83	56.1		129	87.2		133	89.9	
No 6. (Płock) (*n* = 319)	140	43.9		237	74.3		283	88.7		300	94.0	
No 7. (Radom) (*n* = 335)	135	40.3		176	52.5		288	86.0		290	86.6	
No 8. (Siedlce) (*n* = 170)	78	45.9		60	35.3		158	92.9		147	86.5	
No 9. (Żyrardów) (*n* = 177)	82	46.3		92	52.0		155	87.6		149	84.2	

Note: statistically significant variables are bolded.

In multivariable logistic regression, females compared to males were more likely to declare visiting an ophthalmologist in the last 2 years (*p =* 0.008), but also experiencing difficulties accessing an ophthalmologist (*p <* 0.001). Respondents with higher education were more likely to visit ophthalmologists in the last 2 years (*p =* 0.003). Moreover, those taking medications on a regular basis (*p =* 0.02) were also more likely to visit ophthalmologists in the last 2 years (Table 6). Odds of expecting difficulties accessing an ophthalmologist increased with the age group (Table 6).

## 4. Discussion

This epidemiological study provides detailed characteristics of participants of one of the largest opportunistic screenings on eye diseases, implemented by a local government unit in Poland. Findings from this study showed that implementation of the program led to the detection of 38 cases of glaucoma (2.1% of all participants), 84 new cases of AMD (4.6% of all participants), and 21 cases of diabetic retinopathy (1.2% of all participants). Most of the participants of the opportunistic screening program for eye diseases had not visited ophthalmologists in the last 2 years (58.6%), declared low or average level of knowledge on eye health, had difficulty accessing an ophthalmologist in their region (57%) and indicated long waiting time for a visit as a major barrier to accessing eye care (83.5%). A significantly higher detection rate of eye diseases was noticed among those with older age, a lack of higher education, and those taking medications daily. Moreover, socio-demographic barriers to access to eye care services were described.

Population aging in Poland significantly contributes to the growing burden of eye diseases [23]. However, despite the universal healthcare coverage and mandatory health insurance in Poland, barriers to accessing health services, including eye care services, are still present [6,24]. Community initiatives on early detection of eye conditions may pose a significant support for the national health system and limit the number of underdiagnosed cases of eye diseases. However, between 2015 and 2023, eye health and screening for eye conditions were the scope of 41 (2.6% of all programs) LGUs’ health policy programs in Poland. There is a limited amount of scientific data on local government programs on eye care and screening.

This study provides comprehensive data on one of the biggest LGUs’ programs on opportunistic screening for eye diseases in Poland, which was targeted at populations that may experience barriers to accessing eye care in Mazovian Voivodeship [18]. Findings from this study revealed a relatively high detection rate of AMD (84 new cases; 4.6% of all participants), and a significant number of participants diagnosed with glaucoma (38 new cases; 2.1% of all participants) or diabetic retinopathy (21 new cases; 1.2% of all participants). In total, out of 1812 participants, 143 newly diagnosed cases of eye disease were detected. This finding indicates the screening yield of this kind of opportunistic screening program.

Subjects reported for the opportunistic screening program self-declared on admission their health conditions, including eye diseases diagnosed by a doctor in the past. Among those who participated in screening, 38 participants reported a prior diagnosis of glaucoma that was not confirmed during the screening tests performed in this study. This observation may result from misclassification. However, this observation may also partially result from the low level of awareness of eye diseases among adults in Poland [9,20] and points out the need to perform educational campaigns on eye diseases, especially those addressing glaucoma.

Older age is a significant risk factor for eye diseases (especially AMD) [25]. In this study, the detection rate of AMD and diabetic retinopathy was higher in older individuals, which is in line with the global literature on the pathogenesis of these eye conditions [1,2,3,4]. Older adults more often declared difficulty accessing an ophthalmologist in their region of living. This may result from barriers to accessing healthcare, including transportation barriers, economic barriers, and health literacy-related issues [26].

Females more often declared regular eye check-ups, but also more often had trouble accessing an ophthalmologist in their region of living. Previously published data on the frequency of eye examinations reported a lack of socio-economic differences in the frequency of eye examinations [8]. This observation requires further investigation.

There were significant differences by educational level. The detection rate of AMD and diabetic retinopathy was higher among those without higher education. Moreover, individuals without higher education less frequently declared visiting ophthalmologists in the last 2 years and declared a lack of access to an optician’s shop in their place of residence. This observation underlines the role of targeting preventive programs on eye health to those groups without higher education [27].

In this study, there were also differences in attitudes and behaviors on visiting ophthalmologists and eye health by taking medications regularly. However, due to the lack of data on the types of medication used by participants, this study requires further investigation in more comprehensive studies. Moreover, there were differences in the detection rate of eye conditions and perception of barriers to access eye care by research site, which pointed out health inequalities in eye care by place of residence. This observation also supports the rationale for this kind of opportunistic screening program organized by the local government units [15,16,17].

In response to the growing burden of eye conditions, currently in Poland, there is a debate in place on the role of opticians and optometrists in eye health screening [7,28]. Opticians are technical professionals responsible for dispensing and fitting optical appliances (spectacles and contact lenses) based on a prescription issued by an ophthalmologist or certified optometrist [28]. Optometrists are university-trained specialists (medical professionals under the Polish law), tasked with vision care, refraction assessment, assessment of binocular vision and accommodation, contact lens fitting, and screening for ocular pathology [28]. Ophthalmologists are medical doctors providing a full scope of eye care (including prevention, ambulatory care, and surgical procedures). In practice, patients with vision impairment visit optometrists to assess refraction and fit the spectacles or contact lenses. Eye conditions and diseases, like AMD, glaucoma, and diabetic retinopathy, are treated by ophthalmologists. From June 2025, optometrists have acquired additional competencies and can independently perform several tests and conduct educational and preventive activities [29]. In the new model of eye care that is being implemented in Poland, patients will be evaluated by the optometrist and referred to an ophthalmologist, mostly for threatening situations or surgeries [7].

This study has practical implications for policymakers at both national and international levels. This study pointed out that opportunistic screening for eye diseases in community settings may be characterized by relatively high screening yield. The high detection rate of AMD suggests that this kind of preventive action undertaken by local government units may be implemented in populations with limited access to eye care services. Moreover, this study also revealed that older age and lack of higher education are important socio-demographic factors that should be considered during the identification of priority populations and target groups for the health programs on opportunistic screening for eye diseases. The current healthcare system in Poland at the national level does not provide a sufficient number of public health interventions aimed at early detection of eye diseases causing irreversible visual impairment, so local government units may address these issues, and further actions are needed to provide more comprehensive eye care (especially early diagnosis) in the healthcare system in Poland.

### Limitations

This study is a retrospective analysis of data collected during the Local Government Unit program on opportunistic screening for eye diseases, “Good Vision for Mazovians”. The scope of analysis is limited to data available in the medical records collected by the ophthalmological team. This was an opportunistic screening, so the population that took part in this study was not representative, and self-selection bias should be noted. In this study, outcomes related only to 3 eye diseases (glaucoma, AMD, and diabetic retinopathy) were described and analyzed. Data on medical conditions diagnosed in the past and medications taken on a regular basis were self-reported, and no medical records were reviewed on admission, so there is a risk of recall bias. There were no available data on post-screen confirmation rates, so detection rates should be interpreted carefully. Short duration (12 screening days) may limit seasonal representativeness.

## 5. Conclusions

This study showed that opportunistic screening for eye diseases targeted to populations with limited access to eye care may be considered as a method to identify common causes of irreversible visual impairment, especially AMD. Older adults and those without higher education are sociodemographic groups that may face the highest barriers to accessing eye health and may benefit the most from opportunistic screening programs.

## Figures and Tables

**Table 1:** Characteristics of the study population (*n* = 1812).

Variable	*n*	%
Gender		
Female	1081	59.7
Male	731	40.3
Age		
<30	73	4.0
30–39	94	5.2
40–49	292	16.1
50–59	291	16.1
60–69	513	28.3
70–79	465	25.7
80+	84	4.6
Educational level		
Primary	108	6.0
Vocational	370	20.4
Secondary	715	39.5
Higher	619	34.2
Taking medications on a regular basis		
Yes	1137	62.7
No	675	37.3
Has a doctor ever diagnosed you with any chronic diseases?		
Hypertension	748	41.3
Heart disease	311	17.2
Lung disease	120	6.6
Diabetes	213	11.8
Do you smoke cigarettes, use e-cigarettes, or heated tobacco?		
Yes	306	16.9
No	1506	83.1
Research site		
No 1. (Bodzanów)	159	8.8
No 2. (Ciechanów)	289	15.9
No 3. (Wilanów District)	107	5.9
No 4. (Legionowo)	108	6.0
No 5. (Ostrołęka)	148	8.2
No 6. (Płock)	319	17.6
No 7. (Radom)	335	18.5
No 8. (Siedlce)	170	9.4
No 9. (Żyrardów)	177	9.8

**Table 2:** Social perception of access to eye care and history of eye examinations among the inhabitants of Mazovia, Poland (*n* = 1812).

Variable	*n*	%
Do you wear glasses or contact lenses?		
Yes	1081	59.7
No	731	40.3
Have you ever been diagnosed with chronic eye diseases?		
Glaucoma	69	3.8
AMD	13	0.7
Diabetic retinopathy	11	0.6
When did you last perform an eye examination?		
In the last month	35	1.9
More than a month ago, but less than 12 months ago	335	18.5
More than a year ago, but less than 2 years ago	380	21.0
More than 2 years ago, but less than 3 years ago	280	15.5
More than 3 years ago	645	35.6
Never	137	7.6
Have you visited an ophthalmologist in the last 2 years?		
Yes	750	41.4
No	1062	58.6
Do you have difficulty accessing an ophthalmologist in your region?		
Yes	1033	57.0
No	779	43.0
What problems with access to an ophthalmologist have you noticed in your place of residence? (*n* = 1033)		
Waiting time for a visit	863	83.5
Distance of the clinic/ophthalmologist from the place of residence	79	7.6
Transportation to the medical facility	35	3.4
Cost of private visits to the ophthalmologist	327	31.7
Lack of qualified ophthalmologist in the place of residence	95	9.2
In your place of residence (e.g., city, municipality), is there access to an optician’s shop where you can buy glasses or contact lenses?		
Yes	1511	83.4
No	301	16.6
How would you rate your level of knowledge about eye diseases?		
Very low	281	15.5
Rather low	496	27.4
Average	963	53.1
Rather high	62	3.4
Very high	10	0.6
Would you like to improve your knowledge about eye diseases?		
Yes	1588	87.6
No	224	12.4

**Table 3:** The detection rate of eye conditions among participants of the local government health policy program in Mazovia, Poland (*n* = 1812).

		Self-Declared Prior Medical Diagnosis of Eye Conditions (Before Participation in Screening)	Cases Suspected During the Examination	Newly Suspected Cases	Detected Cases That Are Consistent with the Previous Diagnosis	Cases Declared by the Patient but Not Detected During the Examination Through Screening
Glaucoma	*n*	69	69	38	31	38
	% (95%CI)	3.8 (3.0–4.8)	3.8 (3.0–4.8)	2.1	1.7	2.1
AMD	*n*	13	92	84	8	5
	% (95%CI)	0.7 (0.4–1.2)	5.1	4.6	0.4	0.3
Diabetic retinopathy	*n*	11	29	21	8	3
	% (95%CI)	0.6 (0.3–1.1)	1.6	1.2	0.4	0.2

**Table 4:** Socio-demographic characteristics of newly detected suspected cases of ophthalmological diseases.

	Newly Detected Suspected Cases of Ophthalmological Disease											
	Glaucoma				AMD				Diabetic Retinopathy			
Variable	*n*	%	95%CI	*p*	*n*	%	95%CI	*p*	*n*	%	95%CI	*p*
Gender												
Female (*n* = 1081)	22	2.0	1.4–3.0	0.8	55	5.1	3.9–6.6	0.3	16	1.5	0.9–2.4	0.1
Male (*n* = 731)	16	2.2	1.4–3.5		29	4.0	2.8–5.6		5	0.7	0.3–1.6	
Age												
<30 (*n* = 73)	0	0.0	0.0–5.0	0.7	0	0.0	0.0–5.0	**<0.001**	0	0.0	0.0–5.0	**<0.001**
30–39 (*n* = 94)	1	1.1	0.2–5.8		1	1.0	0.2–5.8		0	0.0	0.0–3.9	
40–49 (*n* = 292)	6	2.1	1.0–4.4		2	0.7	0.2–2.5		0	0.0	0.0–1.3	
50–59 (*n* = 291)	4	1.4	0.5–3.5		5	1.7	0.7–4.0		1	0.3	0.1–1.9	
60–69 (*n* = 513)	12	2.3	1.3–4.0		13	2.5	1.5–4.3		4	0.8	0.3–2.0	
70–79 (*n* = 465)	13	2.8	0.3–2.2		43	9.2	6.9–12.2		12	2.6	1.5–4.5	
80+ (*n* = 84)	2	2.4	0.7–8.3		20	23.8	16.0–33.9		4	4.8	1.9–11.6	
Educational level												
Primary (*n* = 108)	3	2.8	1.0–7.9	0.2	11	10.2	5.8–17.3	**0.007**	4	3.7	1.5–9.1	**0.02**
Vocational (*n* = 370)	12	3.2	1.9–5.6		23	6.2	4.2–9.2		7	1.9	0.9–3.9	
Secondary (*n* = 715)	15	2.1	1.3–3.4		27	3.8	2.6–5.4		4	0.6	0.2–1.4	
Higher (*n* = 619)	8	1.3	0.7–2.5		23	3.7	2.5–5.5		6	1.0	0.4–2.1	
Taking medications on a regular basis												
Yes (*n* = 1137)	25	2.2	1.5–3.2	0.7	67	5.9	4.7–7.4	**<0.001**	21	1.8	1.2–2.8	**<0.001**
No (*n* = 675)	13	1.9	1.1–3.3		17	2.5	1.6–4.0		0	0.0	0.0–0.6	
Do you smoke cigarettes, use e-cigarettes, or heated tobacco?												
Yes (*n* = 306)	8	2.6	1.3–5.1	0.5	12	3.9	2.3–6.7	0.5	4	1.3	0.5–3.3	0.8
No (*n* = 1506)	30	2.0	1.4–2.8		72	4.8	3.8–6.0		17	1.1	0.7–1.8	
Research site												
No 1. (Bodzanów) (*n* = 159)	6	3.8	1.7–8.0	**<0.001**	8	5.0	2.6–9.6	**<0.001**	1	0.6	0.1–3.5	0.7
No 2. (Ciechanów) (*n* = 289)	2	0.7	0.2–2.5		11	3.8	2.1–6.7		3	1.0	0.4–3.0	
No 3. (Warsaw–Wilanów District) (*n* = 107)	3	2.8	1.0–7.9		18	16.8	10.9–25.0		1	0.9	0.2–5.1	
No 4. (Legionowo) (*n* = 108)	0	0.0	0.0–3.4		0	0.0	0.0–3.4		0	0.0	0.0–3.4	
No 5. (Ostrołęka) (*n* = 148)	0	0.0	0.0–2.5		5	3.4	1.5–7.7		4	2.7	1.1–6.7	
No 6. (Płock) (*n* = 319)	4	1.3	0.5–3.2		15	4.7	2.9–7.6		4	1.3	0.5–3.2	
No 7. (Radom) (*n* = 335)	7	2.1	1.0–4.3		13	3.9	2.3–6.5		3	0.9	0.3–2.6	
No 8. (Siedlce) (*n* = 170)	0	0.0	0.0–2.2		3	1.8	0.6–5.1		2	1.2	0.3–4.2	
No 9. (Żyrardów) (*n* = 177)	16	9.0	5.6–14.2		11	6.2	3.5–10.8		3	1.7	0.6–4.9	

Note: statistically significant variables are bolded.

**Table 6:** Factors associated with the use of eye care services—multivariable logistic regression (*n* = 1812).

Factors Associated with the Use of Eye Care Services—Multivariable Logistic Regression						
	Have You Visited an Ophthalmologist in the Last 2 Years?—“Yes”			Do you Have Difficulty Accessing an Ophthalmologist in Your Region?—“Yes”		
Variable	OR	95%CI	*p*	OR	95%CI	*p*
Gender						
Female	1.31	1.07–1.59	**0.008**	1.65	1.36–2.01	**<0.001**
Male	Reference			Reference		
Age						
<30	Reference			Reference		
30–39	0.37	0.20–0.71	**0.003**	2.33	1.17–4.65	**0.02**
40–49	0.43	0.25–0.74	**0.002**	3.15	1.73–5.71	**<0.001**
50–59	0.43	0.25–0.73	**0.002**	3.73	2.05–6.77	**<0.001**
60–69	0.60	0.36–0.99	**0.04**	5.51	3.08–9.86	**<0.001**
70–79	0.80	0.48–1.33	0.4	4.71	2.62–8.47	**<0.001**
80+	1.07	0.56–2.05	0.8	5.86	2.84–12.07	**<0.001**
Educational level						
Primary	Reference			Reference		
Vocational	1.06	0.67–1.66	0.8	0.99	0.62–1.56	0.9
Secondary	1.14	0.74–1.75	0.6	0.92	0.60–1.43	0.7
Higher	1.93	1.25–3.00	**0.003**	0.85	0.55–1.33	0.5
Taking medications on a regular basis						
Yes	1.29	1.04–1.61	**0.02**	1.23	0.99–1.53	0.06
No	Reference			Reference		

Note: statistically significant variables are bolded.

## Data Availability

The data presented in this study are available on request from the corresponding author due to internal regulations.
